# Biological health or lived health: which predicts self-reported general health better?

**DOI:** 10.1186/1471-2458-14-189

**Published:** 2014-02-21

**Authors:** Cristina Bostan, Cornelia Oberhauser, Gerold Stucki, Jerome Bickenbach, Alarcos Cieza

**Affiliations:** 1 Department of Health Sciences and Health Policy, University of Lucerne, Lucerne, Switzerland; 2 Swiss Paraplegic Research, Nottwil, Switzerland; 3 Department of Medical Informatics, Biometry and Epidemiology – IBE, Public Health and Health Services Research, Research Unit for Biopsychosocial Health, Ludwig-Maximilians-University (LMU), Munich, Germany; 4 Faculty of Social and Human Sciences, School of Psychology, University of Southampton, Southampton, UK

**Keywords:** Self-reported general health, Biological health, Lived health, Graded Response Model, Random Forest, Spain

## Abstract

**Background:**

Lived health is a person’s level of functioning in his or her current environment and depends both on the person’s environment and biological health. Our study addresses the question whether biological health or lived health is more predictive of self-reported general health (SRGH).

**Methods:**

This is a psychometric study using cross-sectional data from the Spanish Survey on Disability, Independence and Dependency Situation. Data was collected from 17,739 people in the community and 9,707 from an institutionalized population. The following analysis steps were performed: (1) a biological health and a lived health score were calculated for each person by constructing a biological health scale and a lived health scale using Samejima’s Graded Response Model; and (2) variable importance measures were calculated for each study population using Random Forest, with SRGH as the dependent variable and the biological health and the lived health scores as independent variables.

**Results:**

The levels of biological health were higher for the community-dwelling population than for the institutionalized population. When technical assistance, personal assistance or both were received, the difference in lived health between the community-dwelling population and institutionalized population was smaller. According to Random Forest’s variable importance measures, for both study populations, lived health is a more important predictor of SRGH than biological health.

**Conclusions:**

In general, people base their evaluation of their own health on their lived health experience rather than their experience of biological health. This study also sheds light on the challenges of assessing biological health and lived health at the general population level.

## Background

Self-reported general health (SRGH) is the most widely used measure of health in both population and clinical health surveys and the most frequent tool for health comparisons between populations. A Medline literature search showed that in the year 2002, 1,991 scientific papers were published using this question [1]. Most of these studies relied on the standard ‘In general, how would you rate your health?’ question answered on a five-point Likert-type scale: very bad, bad, fair, good, very good, or poor, fair, good, very good, excellent. This question is also included in widely-used questionnaires, such as the Short-Form 36 [2] and the European Organization for Research and Treatment of Cancer Quality of Life Questionnaire [3].

The studies using SRGH usually belong to one of two types: SRGH is used either as a predictor of specific health outcomes, such as mortality [4,5], social-psychological well-being [6,7], morbidity [8,9] and health care utilisation [10], or as an outcome of other factors such as medical diagnoses, physical symptoms and functioning [11], social role activities, social relationships [12] and emotional factors [13].

SRGH is not, however, an approach to measuring health that fits all purposes. Salomon et al*.*[14] claim that SRGH may not be suitable for tracking changes in population health over time and for comparing the level of health of subpopulations.

We claim in this paper that one reason to question the validity of SRGH for tracking health over time and for cross-population comparability involves the different meanings of health that respondents have in mind when answering the SRGH question.

To test what respondents have in mind when answering the SRGH question, qualitative studies are a good place to begin. These studies scrutinize what respondents are thinking about when answering. Some of these studies have already shown that SRGH is a multidimensional construct and that the perception of health is determined not only by the presence or the absence of health problems (that is, biological health), but also by one or more of the following factors: (1) functional factors - the extent to which people are able to perform actions and tasks; (2) coping factors - the person’s level of adaptability, or his or her attitudes towards the health condition, and (3) wellbeing factors - their emotions or feelings [15]. These qualitative studies also suggest that it is very important to anchor the assessment of SRGH to age, gender and time [15,16].

Bearing in mind the value of these studies, the question we wish to answer in this investigation is whether we can psychometrically study what respondents have in mind when answering the SRGH question. To address this question we will use the conceptual basis of the International Classification of Functioning, Disability and Health (ICF) of the World Health Organization (WHO) [17]. According to the ICF model the construct of capacity reflects the intrinsic features of a person to do an action or execute a task independent of the positive or negative influence of the person’s physical, attitudinal or social environment. The construct of performance, on the other hand, refers to health in terms of what one’s level of capacity in different functioning domains allows us to do in life, taking full account of the impact, positive or negative of one’s environment, such as the assistive devices one may use. Health in the sense of capacity is what we mean by ‘biological health’ and performance is what we mean by ‘lived health’. The ICF provides the best framework to describe and measure people’s limitations and restrictions and was explicitly not intended to measure quality of life understood as how people feel about these limitations and restrictions.

For this investigation, we selected a population-based study, namely the 2008 Spanish National Disability Survey. We selected this study because it captured both the concepts of biological health and lived health, making it possible for us answer the question whether SRGH is more related to one or the other. The questionnaires used for this survey contained the SRGH question as well as questions about the extent of problems in different domains of functioning, with and without assistive devices or personal assistance. We believe that the extent of problems in domains of functioning without any aids or personal assistance captures biological health, whereas questions about the extent of problems in the same domains but taking into consideration personal or technical assistance addresses the concept of lived health. The aim of this study is, again, to determine whether biological health or lived health is more predictive of SRGH.

## Methods

### Study design and participants

This is a psychometric study using cross-sectional data from the Spanish National Disability Survey from 2008 (Survey on Disabilities, Independence and Dependence Situations - EDAD). This survey included two residence-based population samples, one community-dwelling and the other institutionalized. The 2008 EDAD design has been described previously [18]. Data was only collected for people who fulfilled the disability criterion of having ‘important limitations to carrying out everyday activities that have lasted, or are expected to last, more than one year, and whose origin is an impairment in one of the following eight domains: *seeing, hearing, communication, learning and application of knowledge and development of tasks, mobility, self-care, home life, interactions and interpersonal relationships*.

### Variables

Forty-two questions were used to assess the level of difficulty in carrying out activities without any technical aid or personal assistance. In our judgment, these are questions about a person’s biological health. Thirty-one questions assessing the level of difficulty in most of the same activities but taking into account any kind of technical aid or personal assistance were also asked. These we judged to be lived health questions. The ordinal scale used to assess the limitation level consisted of the following response options: 1 = Without difficulty or with little difficulty; 2 = With moderate difficulty; 3 = With severe difficulty and 4 = Cannot carry out the activity.

When people did not use technical assistance or have personal assistance, only the question about the level of difficulty ‘without’ was asked. Additional questions about medical conditions, diagnosis, professional life, education, discrimination, social contacts, accessibility and main caregivers were also asked. The SRGH level was collected using the five point scale, with response options: very bad, bad, fair, good, very good.

### Data analysis

The questions referring to vision and hearing were not considered because no differentiation was made between with and without assistive devices or personal assistance. Furthermore, only people that had a difficulty in at least one of the remaining biological health questions were included in the analyses. As a result, 17,739 people from the community-dwelling and 9,707 from the institutionalized population were kept in the analyses.

We used descriptive statistics to present the characteristics of both study populations, taking sampling weights into account. The response options ‘With moderate difficulty’ and ‘With severe difficulty’ in both biological health and lived health questions showed a low frequency. Thus, we collapsed them into a single option called ‘with moderate/severe difficulty’.

To answer the question whether biological health or lived health is more predictive of SRGH, we (1) calculated a biological health and a lived health score for each person by constructing a biological health scale (BHS) and a lived health scale (LHS) using the Item Response Theory (IRT) Model called Samejima’s Graded Response Model (GRM); and (2) calculated the variable importance measures using Random Forest with SRGH as the dependent variable and the biological health score and lived health score as independent variables.

For step one, three specific steps were followed:

a) We evaluated the assumptions of Item Response Theory (IRT) - unidimensionality, local independency and monotonicity - separately for biological health and lived health questions to find out whether IRT could be used for our data. Unidimensionality was examined with bifactor analysis with the analytic bifactor rotations [19,20]. Local independency was tested by examining the residual correlations among questions in one-factor model confirmatory factor analysis [21]. We estimated GRM with and without the flagged local dependent questions (residual correlations higher than 0.2) to see if results were robust to question dependencies [22]. Monotonicity was studied by examining graphs of the question mean scores conditional on ‘rest-scores’ (i.e. total raw scale score minus the question score). Questions that failed one of these three assumptions were not considered in the final model [23].

b) Biological health questions and lived health questions that satisfied the IRT assumptions were used to create a BHS and a LHS using GRM [24].

c) Biological health questions and lived health questions were tested for differential item functioning (DIF) for study population (institutionalized and community-dwelling), gender (male and female), age groups (≤65 and >65) and reported number of health conditions groups (0, 1-2 and >2) using iterative hybrid ordinal logistic regression with change in McFadden’s pseudo R-squared measure (above 0.02) as DIF criterion [25,26]. Questions showing DIF were calibrated separately for each of the groups showing DIF and after DIF correction final GRMs were calculated. Based on the resulting biological health question parameters and lived health question parameters, a summary score of biological health and a summary score of lived health for each of the individuals in the sample were calculated. For a more intuitive summary score for the biological health or lived health of individuals, we transformed the resulting scores into more meaningful values, ranging from 0 (best biological health or lived health) to 100 (worst biological health or lived health). For both study populations, the relation between biological health and lived health was studied using the Pearson correlation analysis.

For step two, for each of the community-dwelling and institutionalized data sets, we (1) studied the association between biological health scores, lived health scores and SRGH by using Spearman correlation coefficient (r_S_) and box-plots which displayed the distribution of biological health scores and lived health scores in each of the five SRGH response options; and (2) compared the importance value of the biological health score with that of the lived health score obtained from Random Forest regression with 1000 trees and mtry = 2, where 2 means the number of randomly preselected independent variables, which in Random Forest are called split variables. The Random Forest regression provides an improved prediction accuracy compared to other regression techniques (e.g. logistic or linear regression) because it deals with the collinearity and the main and interaction effects of independent variables. The variable importance measure is the average of the frequency with which the independent variables (biological health and lived health) appear in all 1000 trees calculated to predict the dependent variable (SRGH) over all 1000 trees. It takes values from 0 to 1, the higher the value, the better the prediction of SRGH. The permutation importance was computed with the conditional permutation scheme proposed by Strobl and colleagues, which controls for the correlation of the predictor variables [27].

All the analyses were performed with R version 2.15.1 [28].

## Results

Characteristics of both study populations are presented in Table 1. In both study populations around 60% were female. Most of the institutionalized people were aged more than 65 years (82%). The percentage of respondents reporting very good or good health is 38.2 in the institutionalized and 20.7 in the community-dwelling population.

**Table 1:** Characteristics of institutionalized and community-dwelling population

	**Institutionalized population**	**Community-dwelling population**
	**(N = 9707)**	**(N = 17739)**
	**%**	**%**
Gender		
Female	65.5	63.3
Age, years		
Old (>65)	82.0	58.5
Education		
No school	21.8	11.9
Primary-school-incomplete	45.3	35.6
Primary-school-complete	23.4	28.8
Secondary school first step	3.0	9.3
Secondary school finished	3.1	5.3
Professional school medium	1.3	2.8
Professional school superior	0.5	1.7
University	1.5	4.6
SRGH		
SRGH: very good	3.0	1.5
SRGH: good	35.2	19.2
SRGH: fair	37.1	44.9
SRGH: bad	18.8	26.5
SRGH: very bad	5.9	7.9
Number of health conditions^a^		
No health condition	12.3	12.4
One or two health conditions	68.6	50.1
Three or more than three health conditions	19.1	37.5

All data are population weighted.

SRGH = Self-reported general health.

^a^The health conditions were: Spinal cord injury, Parkinson’s, Lateral sclerosis, Multiple sclerosis, Agenesis/Amputation, Laryngectomy, Arthritis, Rheumatoid arthritis or Ankylosing spondylitis, Muscular dystrophy, Spina bifida/hydrocephaly, Myocardial infarction or Ischaemic cardiopathy, Cerebrovascular accidents, Down's Syndrome, Autism and other disorders associated with autism, Cerebral paralysis, Acquired brain damage, Senile Dementia of the Alzheimer Type, Other types of dementia, Schizophrenia, Depression, Bipolar disorder, Pigmentary retinosis, Myopia magna, Senile macular degeneration, Diabetic retinopathy, Glaucoma, Cataract, HIV/AIDS, Rare illnesses, Cancer (only for community-dwelling population).

Table 2 shows the biological health and lived health questions considered for BHS and LHS, respectively.

**Table 2:** Biological health and lived health questions initially considered for the GRM models for each study population: institutionalized and community-dwelling population

	**Biological health scale**					**Lived health scale**				
**Questions**	**Variable**	**Institutionalized population**		**Community-dwelling population**		**Variable**	**Institutionalized population**		**Community-dwelling population**	
		**N**	**%difficulty**	**N**	**%difficulty**		**N**	**%difficulty**	**N**	**%difficulty**
**Communication**										
With what level of difficulty would you say you are able to…										
Speak intelligibly or utter coherent phrases?	COM_8_2	9702	36.4	15912	14.8	**COM_8_3b**^**6**^	9696	36.1	15912	14.7
Understand what other persons say to you?	COM_9_2	9703	35.6	15671	13.4	COM_9_3b^6^	9688	32.9	15661	12.1
Understand and express yourself in writing?	COM_10_2	9687	47.6	16115	16.0					
Understand and express yourself via gestures, symbols, illustrations or sounds?	COM_11_2	9703	34.8	15048	9.8					
Hold a dialogue and exchange ideas with one or more persons?	COM_12_2	9698	41.6	15899	14.8					
Use the telephone or other devices or communication techniques?	COM_13_2	9678	51.4	16605	18.0	COM_13_3b^6^	9666	45.4	16578	15.8
**Learning and application of knowledge and development of tasks**										
With what level of difficulty are you able to…										
Hold a gaze or pay attention when listening?	APR_14_2	9705	29.2	15793	10.2					
Learn to perform simple tasks?	APR_15_2	9665	41.2	16657	14.9					
Perform simple tasks?	APR_16_2	9707	34.0	16068	11.4	**APR_16_3b**^**7**^	9680	29.9	16054	10.0
Perform complex tasks?	APR_17_2	9707	49.7	17229	17.6	**APR_17_3b**^**7**^	9676	44.2	17208	15.2
**Mobility**										
With what level of difficulty are you able to…										
Change posture?	MOV_18_2^1^	9698	45.9	9578	64.6	MOV_18_3b^8^	9599	36.3	9527	55.3
Keep the body in the same position?	MOV_19_2^1^	9704	41.2	9923	66.1	MOV_19_3b^8^	9645	33.5	9869	59.6
Walk and move around the home?	**MOV_20_2**^**1**^	9705	53.4	8751	61.0	**MOV_20_3b**^**8**^	9641	40.3	8701	53.0
Walk or move outside the home?	**MOV_21_2**^**2**^	9683	81.4	12919	74.3	**MOV_21_3b**^**9**^	9640	62.5	12846	64.1
Get around via passenger transport?	MOV_22_2^2^	9693	85.7	12156	73.2	MOV_22_3b^9^	9649	68.5	12079	63.4
Drive vehicles?	MOV_23_2^2^	3653	82.7	4378	55.5	MOV_23_3b	3648	82.1	4378	51.5
Lift or carry objects?	MOV_24_2^3^	9705	52.3	11259	70.6	**MOV_24_3b**^**10**^	9684	47.3	11215	63.7
Handle and move objects?	**MOV_25_2**^**3**^	9705	36.0	9056	63.2	**MOV_25_3b**^**10**^	9685	32.5	9022	57.1
Lift or carry small objects?	MOV_26_2^3^	9704	38.6	7840	56.8	**MOV_26_3b**^**10**^	9682	34.5	7816	51.8
**Self-care**										
With what level of difficulty would you say are you able to…										
Wash or dry different body parts?	AUT_27_2	9702	77.6	14557	49.8	AUT_27_3b	9600	57.1	14491	40.0
Perform basic grooming?	AUT_28_2	9702	78.1	14249	48.9	AUT_28_3b	9611	58.8	14160	38.0
Carry out activities related to urination?	AUT_29_2	9704	52.6	11346	36.4	AUT_29_3b	9657	41.6	11295	30.4
Carry out activities related to defecation?	AUT_30_2	9701	48.3	9979	28.0	AUT_30_3b	9663	38.8	9946	24.6
Carry out activities related to menstrual care?	AUT_31_2	680	56.6	1501	12.1	AUT_31_3b	674	38.0	1501	9.6
Dress or undress?	AUT_32_2	9706	59.5	13035	44.2	AUT_32_3b	9648	45.3	12975	35.2
Eat and drink?	AUT_33_2	9704	31.0	9301	22.9	AUT_33_3b	9690	23.8	9269	18.4
Follow medical prescriptions?	AUT_34_2	9693	69.2	12085	40.2	AUT_34_3b	9627	44.1	12032	24.3
Avoid dangerous situations?	AUT_35_2	9667	62.4	10987	34.4	AUT_35_3b	9621	51.9	10951	27.1
**Home Life**										
With what level of difficulty would you say are you able to…										
Do shopping?	VDOM_36_2^4^	9675	84.8	16405	65.6	**VDOM_36_3b**^**11**^	2355	22.1	16270	54.9
Prepare meals?	VDOM_37_2^4^	9243	84.3	12601	54.3	**VDOM_37_3b**^**11**^	1897	17.8	12523	47.4
Carry out housework?	**VDOM_38_2**^**4**^	9282	83.1	15257	62.8	**VDOM_38_3b**^**11**^	2238	19.3	15139	54.3
**Interaction and interpersonal relations**										
With what level of difficulty would you say are you able to…										
Show to other persons affection, respect or transmit feelings?	**INTER_39_2**^**5**^	9703	27.4	15768	9.4					
Relate to strangers?	**INTER_40_2**^**5**^	9700	43.6	16666	14.2					
Initiate and maintain relations with subordinates, peers or superiors?	INTER_41_2^5^	9701	26.7	16222	12.0					
Initiate and maintain relations with friends, neighbours, acquaintances or colleagues?	**INTER_42_2**^**5**^	9704	33.5	16264	12.3					
Initiate and maintain family relations?	INTER_43_2^5^	9694	26.9	16281	12.5					
Initiate and maintain intimate or sexual relations?	INTER_44_2	9647	67.0	16660	14.6					

Local dependent variables are marked with the same number. The questions considered in the final model are marked in bold. All data are population weighted.

### Biological health and lived health scores

#### IRT Assumptions

##### Unidimensionality

For both BHS and LHS the bifactor analyses supported the assumption of a strong general factor, with all questions loading highly on the general factor. However, questions from the *mobility* domain of LHS and questions from the *communication* domain and *learning and application of knowledge and development of tasks* domain of LHS loaded higher on their respective group factors than on the general factor. We decided to proceed with unidimensional BHS and unidimensional LHS, since these domains are contributing to biological health and lived health, respectively. We checked our decision by estimating the GRMs both with and without *mobility* for BHS and *communication* and *learning and application of knowledge and development of tasks* for LHS and analyzed the correlation between the item thresholds for the two models each. The results showed that our decision did not affect the results.

##### Local independency

While the examination of the residual correlations of biological health questions indicated violation of local independency in five groups of questions, the results for lived health questions revealed violation in six. Table 2 shows the local dependent questions as well as the questions considered in the final models.

##### Monotonicity

The monotonicity IRT assumption was satisfied by most of the biological health and lived health questions, with the exception of the questions: ‘With what level of difficulty would you say are you able to carry out activities related to menstrual care?’ and ‘With what level of difficulty would you say are you able to drive vehicles?’.

##### Differential item functioning

Table 3 presents the biological health and lived health questions included in the BHS and LHS respectively and their parameter estimates (discrimination and threshold parameters) for the final GRM models. While for BHS 6 questions showed DIF for study population and 11 questions for age groups, for LHS 7 questions showed DIF for study population, and 4 for age groups. All questions of the BHS and of the LHS were free of DIF for gender and number of health conditions.

**Table 3:** Biological health and lived health questions included in the single biological health scale and lived health scale and their parameter estimates (discrimination (Discr) and threshold parameters (Thr 1-2)) for the final GRM models

	**Biological health scale**				**Lived health scale**			
**Questions**	**Split in**	**Discr**	**Thr 1**	**Thr 2**	**Split in**	**Discr**	**Thr 1**	**Thr 2**
**Communication**								
With what level of difficulty would you say you are able to…								
Speak intelligibly or utter coherent phrases?	Young	2.106	0.690	1.698	Institutionalized young	1.325	-0.001	1.452
					Institutionalized old	1.634	0.906	2.073
					Community-dwelling young	0.914	1.795	3.807
	Old	2.681	1.011	2.054	Community-dwelling old	1.222	2.110	3.453
Understand what other persons say to you?		2.375	1.025	2.300				
Understand and express yourself in writing?	Young	2.153	0.521	1.147				
	Old	2.271	0.871	1.514				
Understand and express yourself via gestures, symbols, illustrations or sounds?		2.276	1.226	2.010				
Hold a dialogue and exchange ideas with one or more persons?	Young	3.357	0.557	1.422				
	Old	4.291	0.952	1.772				
Use the telephone or other devices or communication techniques?		1.989	0.649	1.427				
**Learning and application of knowledge and development of tasks**								
With what level of difficulty are you able to…								
Hold a gaze or pay attention when listening?		2.066	1.397	2.558				
Learn to perform simple tasks?		1.963	1.043	2.103				
Perform simple tasks?		2.328	1.236	1.940	Institutionalized	2.440	0.800	1.486
					Community-dwelling	1.867	1.745	2.383
Perform complex tasks?		1.871	0.799	1.965	Institutionalized young	1.180	-0.092	1.091
					Institutionalized old	2.018	0.428	1.067
					Community-dwelling	1.346	1.625	2.475
**Mobility**								
With what level of difficulty are you able to…								
Walk and move around the home?	Institutionalized young	2.033	1.433	2.126	Institutionalized young	2.904	1.025	2.172
	Institutionalized old	1.394	0.148	1.427	Institutionalized old	1.957	0.523	2.191
	Community-dwelling	1.987	-0.447	1.196	Community-dwelling	1.390	0.083	2.927
Walk or move outside the home?		0.626	-2.691	1.156		1.813	-0.128	2.097
Lift or carry objects?					Institutionalized	1.938	0.309	1.005
					Community-dwelling	1.314	-0.416	1.731
Handle and move objects?					Institutionalized	2.436	0.800	1.535
					Community-dwelling	1.463	-0.121	2.178
Lift or carry small objects?	Institutionalized	1.895	0.931	1.708	Institutionalized	2.407	0.721	1.576
	Community-dwelling	2.122	-0.319	1.212	Community-dwelling	1.631	-0.034	1.914
**Self-Care**								
With what level of difficulty would you say are you able to…								
Wash or dry different body parts?	Young	2.590	0.019	1.040		2.760	0.197	1.695
	Old	2.475	-0.549	0.908				
Perform basic grooming?		1.922	-0.375	0.994		2.325	0.159	1.597
Carry out activities related to urination?	Institutionalized young	3.571	1.090	1.542		2.562	0.501	1.720
	Institutionalized old	1.728	0.358	1.352				
	Community-dwelling	3.129	-0.169	0.893				
Carry out activities related to defecation?	Young	2.275	0.902	1.682		2.862	0.541	1.637
	Old	2.247	0.373	1.144				
Dress or undress?	Institutionalized young	3.591	0.750	1.545		3.445	0.361	1.565
	Institutionalized old	2.057	0.161	0.923				
	Community-dwelling	3.294	-0.278	0.723				
Eat and drink?	Institutionalized	2.196	1.128	1.914		2.837	0.913	2.165
	Community-dwelling	3.140	-0.043	1.439				
Follow medical prescriptions?		1.789	-0.220	1.875		1.967	0.521	1.648
Avoid dangerous situations?		1.957	0.113	1.457		2.664	0.290	0.955
**Home Life**								
With what level of difficulty would you say are you able to…								
Do shopping?						2.512	-0.062	0.681
Prepare meals?						4.251	0.008	0.543
Carry out housework?		1.152	-1.083	0.645		2.462	-0.099	0.595
**Interaction and interpersonal relations**								
With what level of difficulty would you say are you able to…								
Show to others affection, respect or transmit feelings?	Young	1.820	1.224	2.325				
	Old	2.374	1.423	2.221				
Relate to strangers?		1.837	1.026	1.969				
Initiate and maintain relations with friends, neighbours, acquaintances or colleagues?	Young	1.865	0.952	1.825				
	Old	2.917	1.202	1.806				
Initiate and maintain intimate or sexual relations?	Institutionalized	1.359	-0.175	0.515				
	Community-dwelling young	1.920	0.654	1.325				
	Community-dwelling old	4.970	1.151	1.295				

#### Biological health scale and lived health scale

The most discriminating biological health question was ‘initiate and maintain intimate or sexual relations’ in the community-dwelling old age group (with a discrimination of 4.97). This means that this question differentiates well between people with high and lower difficulties in biological health in the old age group. The least discriminating question was ‘walk or move outside the home’ (with a discrimination of 0.62). For LHS, the most discriminating question was ‘carry out housework’ (with a discrimination of 4.25); the least discriminating was ‘speak intelligibly or utter coherent phrases’ in the community-dwelling young-age group (with a discrimination of 0.91). While the question for which only those individuals in the worst biological health are expected to have median difficulties is ‘hold a gaze or pay attention when listening’ (with a threshold of 2.55 on the logit scale), the question for which individuals in the worst lived health are expected to have high difficulties is ‘speak intelligibly or utter coherent phrases’ in the community-dwelling young-age group (with a threshold of 3.80).

On a scale from 0 (best biological health) to 100 (worst biological health), the levels of biological health are higher for community-dwelling (mean = 31.07, standard deviation = 21.22, range = [0; 98.96]) than for institutionalized population (mean = 48.86, standard deviation = 23.54, range = [0; 100]). When technical assistance, personal assistance or both was received, the difference between community-dwelling (mean = 31.94, standard deviation = 20.72, range = [0; 100]) and institutionalized populations (mean = 36.75, standard deviation = 22.69, range = [2.09; 93.22]) was smaller. The biological health score and lived health score are not comparable since they were calculated based on two separate sets of questions.

For both study populations the Pearson correlation between biological health and lived health was high: 0.79 for community-dwelling population and 0.85 for institutionalized population.

### Conditional permutation importance of biological health and lived health scores

**Figure 1:**
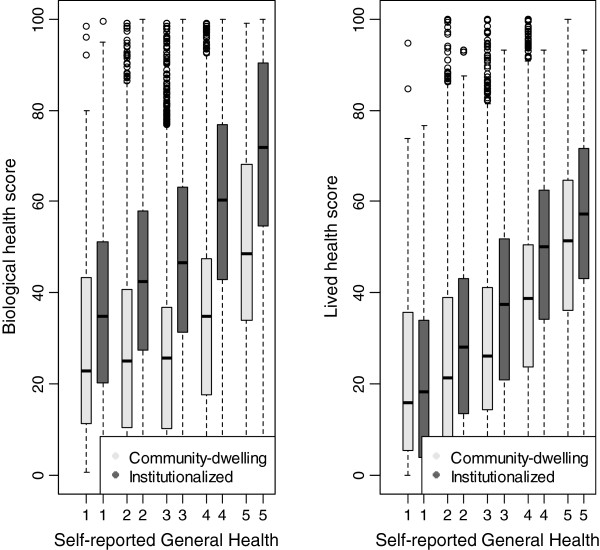
Box-plot showing the distribution of biological health scores and lived health scores in each of the five SRGH response options (1 = very good, 2 = good, 3 = fair, 4 = bad, 5 = very bad).

For both community-dwelling and institutionalized populations the association between SRGH and lived health scores (community dwelling: r_S_ = 0.33, institutionalized: r_S_ = 0.36) was higher than the association between SRGH and biological health scores (community dwelling: r_S_ = 0.23, institutionalized: r_S_ = 0.30). The relation between SRGH and biological health scores and lived health scores is displayed in Figure 1.

**Figure 2:**
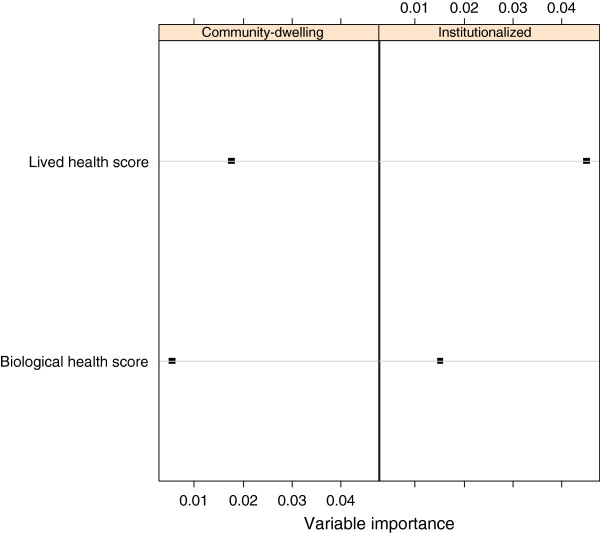
Conditional permutation importance of biological health and lived health scores as predictors of SRGH by study population. The higher the value, the better the prediction of SRGH.

The resulting importance measures of the two predictors (biological health score and lived health score) of SRGH are displayed in Figure 2. For both samples, the lived health score showed the higher variable importance and therefore was a better predictor of SRGH than the biological health score.

## Discussion

Comparing the predictive value for SRGH of biological health and lived health in a psychometric space is the first step towards a true understanding of what people are thinking about when rating their general health. Our study showed that people base their evaluation of their health, not on their biological state, but on their lived experience of their health. This is an important result because it implies that any kind of intervention that targets population health should address, not merely the intrinsic capacity of a person, but also his or her environment.

We are not aware of studies reporting on the comparison of predictive power of biological health and lived health on SRGH. Yet, our finding is similar to, and confirms the Smith et al. [29] conclusion that ‘sickness is a social role in addition to biological state’ and that SRGH ‘is not a continuum of biological states’. As Jylhä [30] suggested, the response to SRGH is influenced not only by ‘earlier health experiences, present health conditions’, but also by the health-related environment.

Bifactor analyses of biological health questions and lived health questions supported the construction of BHS and LHS, in terms of the contribution of questions to a single common dimension. The presence of an underlying factor that links domains of functioning commonly used to operationalize biological health and lived health helped us to quantify both biological and lived health as a single number, which facilitated comparability between people’s abilities from the two study populations. Our results with respect to BHS and LHS are also concordant with other findings [31].

The GRM IRT modelling was used to assess the levels of biological health and lived health. The primary advantage of using an IRT model is that it allows for an estimation of biological health and lived health independent of the set of test questions administrated [32]. For BHS, this makes it possible for us to consider questions that addressed domains of functioning that were not addressed by lived health questions.

The different gradients captured in the developed BHS and LHS - study population and age - support the validity of both scales. However, there is a large number of lived health questions showing DIF. One possible explanation is that institutionalized people receive constant support from hospital personal. This is not the case in the community-dwelling population. In fact more than half of the community-dwelling population did not benefit from personal help. For the age groups, the DIF could be explained by the use of a cut-off of 65 years, which was available in both populations and was in line with others studies that showed that SRGH is worse after an age of 65 years in the Spanish population [33].

For both study populations, the Spearman correlation analysis showed that there is a stronger association between lived health and SRGH than between biological health and SRGH. Since correlation analysis is not a full proof of the strength of biological health and lived health to cause the answer to the SRGH, the regression analysis was used. The causal chain results from the correlation analysis: biological health - > lived health - > SRGH. This implies that linear regression with SRGH as a dependent variable and biological health and lived health as independent variables would have a coefficient zero for biological health, i.e. conditional on lived health, biological health is not contributing anything to predict SRGH. The results of qualitative studies showed that some people will disagree that biological health is unimportant to SRGH, therefore we used the optimal solution of overcoming the structural relation between the variables, namely Random Forest regression and the variable importance measures. Certainly, the causal chain indicates that both biological health and lived health are important factors to consider when people rate their health. However, using the Random Forest regression informs us that it is enough to measure lived health for predicting SRGH.

### Strengths and limitations

The most important strength of this study was its large nationally representative Spanish sample. Yet, it is significant that this sample is only representative of persons with limitations in functioning and not the general population. This is because the design of the 2008 EDAD used a representative Spanish sample as starting point but only obtained more detailed information about lived health from the subpopulation with limitations in biological health. Thus, our results are not generalizable to the entire Spanish population. There were additional limitations. First, more aspects of the environment that affect the experience of health in everyday life should be considered in addition to personal support and technical aids. Secondly, an artificial cut-off was set, in the sense that only what was considered larger than or equal to moderate difficulty could be rated as ‘moderate’, ‘severe’ or ‘cannot carry out the activity’. We had to assume that people answering ‘no difficulty’ were those who either had no or little difficulty, and in any event did not have a severe enough problem to rate it as moderate. We also had to collapse the response options ‘moderate’ and ‘severe’ difficulty because of the skewed distribution towards complete limitation of the response options.

## Conclusions

Our study showed that people base their evaluation of health on their lived health experience rather than their experience of biological health. This result needs to be confirmed and supported by further studies before conclusions can be drawn and practical implications proposed to improve health policy. However, since SRGH can predict the use of health services [34], our study result points to the need on the part of health service personnel and decision makers to consider lived health when they develop and implement health promotion programs or select study outcomes. People with health problems are handed over to health professionals, and this creates an important responsibility. The decisions of health professions should take into account the fact that their patients may be less concerned to know medical facts and more interested in how their health affects everything that they do in their lives. Further research is necessary to determine whether lived health rather that SRGH could be considered when health professionals track health changes over time and for health cross-population comparability.

## Abbreviations

SRGH: Self-reported general health; BHS: Biological health scale; LHS: Lived health scale; GRM: Graded Response Model; IRT: Item Response Theory; DIF: Differential item functioning; EDAD: Survey on disabilities, independence and dependence situations.

## Competing interests

The authors declare that they have no competing interests.

## Authors’ contributions

CB and AC conceived of the study, and its design and drafted the manuscript. CB carried out the statistical analyses. CO made substantial contributions to data analysis. CO, JB and GS critically revised the manuscript in several drafting rounds. All authors read and approved the final manuscript.

## Pre-publication history

The pre-publication history for this paper can be accessed here:

http://www.biomedcentral.com/1471-2458/14/189/prepub
